# Blending of brain-machine interface and vision-guided autonomous robotics improves neuroprosthetic arm performance during grasping

**DOI:** 10.1186/s12984-016-0134-9

**Published:** 2016-03-18

**Authors:** John E. Downey, Jeffrey M. Weiss, Katharina Muelling, Arun Venkatraman, Jean-Sebastien Valois, Martial Hebert, J. Andrew Bagnell, Andrew B. Schwartz, Jennifer L. Collinger

**Affiliations:** Department of Bioengineering, University of Pittsburgh, Pittsburgh, PA USA; Center for the Neural Basis of Cognition, Pittsburgh, PA USA; Department of Physical Medicine and Rehabilitation, University of Pittsburgh, Pittsburgh, PA USA; Robotics Institute, Carnegie Mellon University, Pittsburgh, PA USA; Department of Neurobiology, University of Pittsburgh, Pittsburgh, PA USA; VA Pittsburgh Healthcare System, Pittsburgh, PA USA; University of Pittsburgh, 3520 5th Avenue, Suite 300, Pittsburgh, PA 15213 USA

**Keywords:** Brain-machine interface, Brain-computer interface, Neuroprosthetic, Shared mode control, Assistive technology

## Abstract

**Background:**

Recent studies have shown that brain-machine interfaces (BMIs) offer great potential for restoring upper limb function. However, grasping objects is a complicated task and the signals extracted from the brain may not always be capable of driving these movements reliably. Vision-guided robotic assistance is one possible way to improve BMI performance. We describe a method of shared control where the user controls a prosthetic arm using a BMI and receives assistance with positioning the hand when it approaches an object.

**Methods:**

Two human subjects with tetraplegia used a robotic arm to complete object transport tasks with and without shared control. The shared control system was designed to provide a balance between BMI-derived intention and computer assistance. An autonomous robotic grasping system identified and tracked objects and defined stable grasp positions for these objects. The system identified when the user intended to interact with an object based on the BMI-controlled movements of the robotic arm. Using shared control, BMI controlled movements and autonomous grasping commands were blended to ensure secure grasps.

**Results:**

Both subjects were more successful on object transfer tasks when using shared control compared to BMI control alone. Movements made using shared control were more accurate, more efficient, and less difficult. One participant attempted a task with multiple objects and successfully lifted one of two closely spaced objects in 92 % of trials, demonstrating the potential for users to accurately execute their intention while using shared control.

**Conclusions:**

Integration of BMI control with vision-guided robotic assistance led to improved performance on object transfer tasks. Providing assistance while maintaining generalizability will make BMI systems more attractive to potential users.

**Trial registration:**

NCT01364480 and NCT01894802.

**Electronic supplementary material:**

The online version of this article (doi:10.1186/s12984-016-0134-9) contains supplementary material, which is available to authorized users.

## Background

Recent brain-machine interface (BMI) work has shown that people with tetraplegia can control robotic arms using signals recorded by intracortical electrodes [1–3]. In order to make this technology broadly available to people with upper limb impairment it will need to be reliable under a variety of non-ideal conditions. Intracortical BMIs suffer from limitations that can negatively impact performance including the small number of simultaneously recorded neurons [2, 4], the degradation of recorded signal quality over time [5], and intraday changes in the recorded units [6]. We have recently shown that motor cortex signaling is context-dependent as the extracted signal changes between object grasping and free movement [3]. If this change is not taken into account, the BMI user has limited ability to control the robotic arm near an object. As with natural reaching, the user must determine how to optimally position the hand to grasp the object for the intended action [7]. Currently, BMIs for arm control do not provide somatosensory feedback for the user [1–3], which may impair the normal grasping process [8]. Finally, another potential barrier to optimal performance is that the visual feedback that a BMI user receives is of a robotic arm rather than their own hand, which may introduce sensory conflicts [9].

Intelligent, vision-guided robotic assistance is one way to improve BMI performance during grasping. Specifically, the system could identify objects in the workspace, define stable grasp positions, and stabilize the hand during grasping [10–12]. Previous work towards shared control between a BMI and a computer vision-based system has been limited to specific tasks that were automatically executed by the robot once the user identified a target [13, 14]. Other shared control work with intracortical BMIs has used state switching, such that either the BMI user or the robotic system had control during specific phases [15, 16]. Instead of this ‘hand off’ from volitional to automatic control, a more ideal system would identify the subject’s intention and optimally blend autonomous assistance with the subject’s volition in a seamless fashion. In order to allow the system to generalize across many tasks, it is important that the volitional contribution remains high at all times. Preservation of BMI control over high-level functions such as gross arm movements and target selection would maintain the user’s agency, while automation could handle low-level functions like hand orientation. A generalizable shared control-based BMI should enable the system to operate robustly over a wide variety of functions in different contexts.

The ability to operate a BMI independently for many activities is considered very important by potential BMI users with tetraplegia [17, 18]. Additionally, users of some shared control systems report frustration if it becomes obvious that the system is providing autonomous control, reducing the sense of agency [19]. We propose an alternative approach where the computer provides assistance with low level control tasks such as accurately aligning the hand with an optimal grasp position defined in real-time. The assistance becomes stronger as the system becomes more confident in its identification of the user’s intent. This method of shared control allows the user to make unconstrained movements in the workspace, and then provides assistance once the system deciphers the user’s intent. In this way, arm control can be made more accurate during tasks that require a high degree of accuracy while still allowing the user to directly control the majority of the movement.

This paper describes a shared control system for grasping that improved the ability of 2 subjects with tetraplegia to transfer objects using a BMI to control a robotic arm. The use of shared control also lowered the perceived difficulty of the task. Analysis of the arm movement kinematics during reaches to an object shows that although shared control led to slower peak movement speeds, the resulting trajectories were more stable and efficient. Additionally, we demonstrated the ability of the user to specify their intention through successful completion of a task requiring object selection.

## Methods

### Study design

This study was completed as part of two clinical trials of intracortical BMIs conducted under Investigational Device Exemptions at the University of Pittsburgh [20, 21]. One subject with tetraplegia from each clinical trial participated in this study.

The primary objectives of the study were to determine the extent to which shared control improves the functional performance of a BMI prosthetic arm. Performance was evaluated using reaching and grasping tasks including a clinical assessment, the Action Research Arm Test (ARAT) [22], and a task in which the user was instructed to pick up one of two possible objects, called the multiple object task. The order of shared control and unassisted test blocks were randomized each day and subjects were blinded to order of the blocks.

### Subjects

**Fig. 1:**
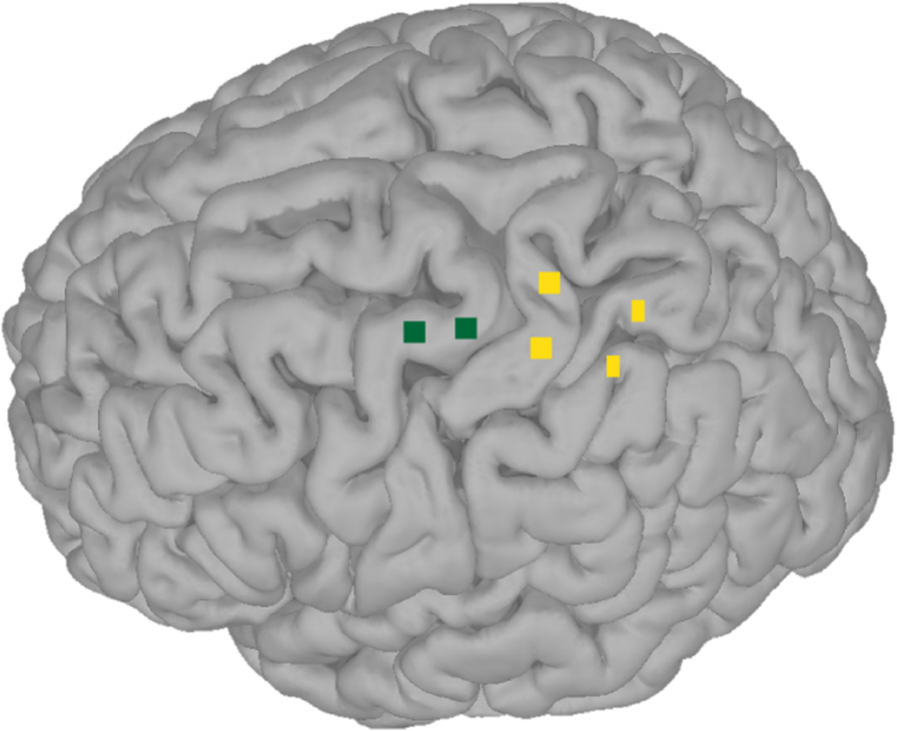
Array location. The approximate location of the microelectrode recording arrays for both subjects on a template brain. Subject 1 had 2 96-channel arrays implanted in M1 (*green squares*). Subject 2 had 2 88-channel arrays implanted in S1 (*yellow squares*) and 2 32-channel arrays implanted more posterior (*yellow rectangles*)

Both subjects provided informed consent prior to participation in any study procedures, which included implantation of intracortical microelectrode arrays (Blackrock Microsystems, Inc., Salt Lake City, Utah). Informed consent was obtained prior to participation in any study procedures. Subject 1 was a 54-year old female diagnosed with spinocerebellar degeneration without cerebellar involvement [23]. Her injury was motor complete at the C4 level, but sensation was generally intact with some hypersensitivity. She had two 4 × 4 mm, 96-channel arrays implanted in her left primary motor cortex (M1; Fig. 1) 31 months prior to the 3 test sessions reported here. Some of the experiments in this study (Multiple Object Task) were developed after Subject 1 was explanted. The study team decided to explant the arrays due to skin retraction around the pedestals. There were no signs of infection, but we decided that explantation was appropriate given the risk/benefit ratio after 987 days in the study. Subject 2 was a 28 year old male with a cervical spinal cord injury. Although a complete examination was not performed, we estimate his injury to be C5 motor and C4 sensory AIS B (www.asia-spinalinjury.org). Subject 2 had two 4 × 4 mm, 88-channel arrays implanted in left somatosensory cortex (S1) and two 4 × 2.4 mm, 32-channel arrays implanted in left parietal cortex (Fig. 1). Subject 2’s arrays were placed relative to anatomical landmarks resulting in placement in somatosensory cortex rather than motor cortex. While this likely contributed to differences in performance between subjects, this placement provided the opportunity to study the benefits of shared control for a BMI using sub-optimal neural control. One session was conducted 7 weeks post-implant and the other two were 14 weeks post-implant. These studies were conducted under Investigational Device Exemptions from the Food and Drug Administration and with approval from the University of Pittsburgh Institutional Review Board.

### Neural recording

Neural data was acquired with the Neuroport Neural Signal Processor (Blackrock Microsystems). At the beginning of each test session a threshold was set for all recorded channels at −4.5 times the root-mean-square voltage (RMS) for Subject 1 or −5.25 times RMS for Subject 2. Firing rates for Subject 1 were estimated for each channel by binning the number of recorded threshold crossings every 30 ms (33 Hz update rate). For Subject 2, a 20 ms bin size was used (50 Hz update rate). Firing rates were low-pass filtered using an exponential smoothing function with a 450 ms window for Subject 1, and a 440 ms window for Subject 2. Each channel was considered to be a neural unit, though many channels recorded multi-unit activity.

### BMI decoding

**Fig. 2:**
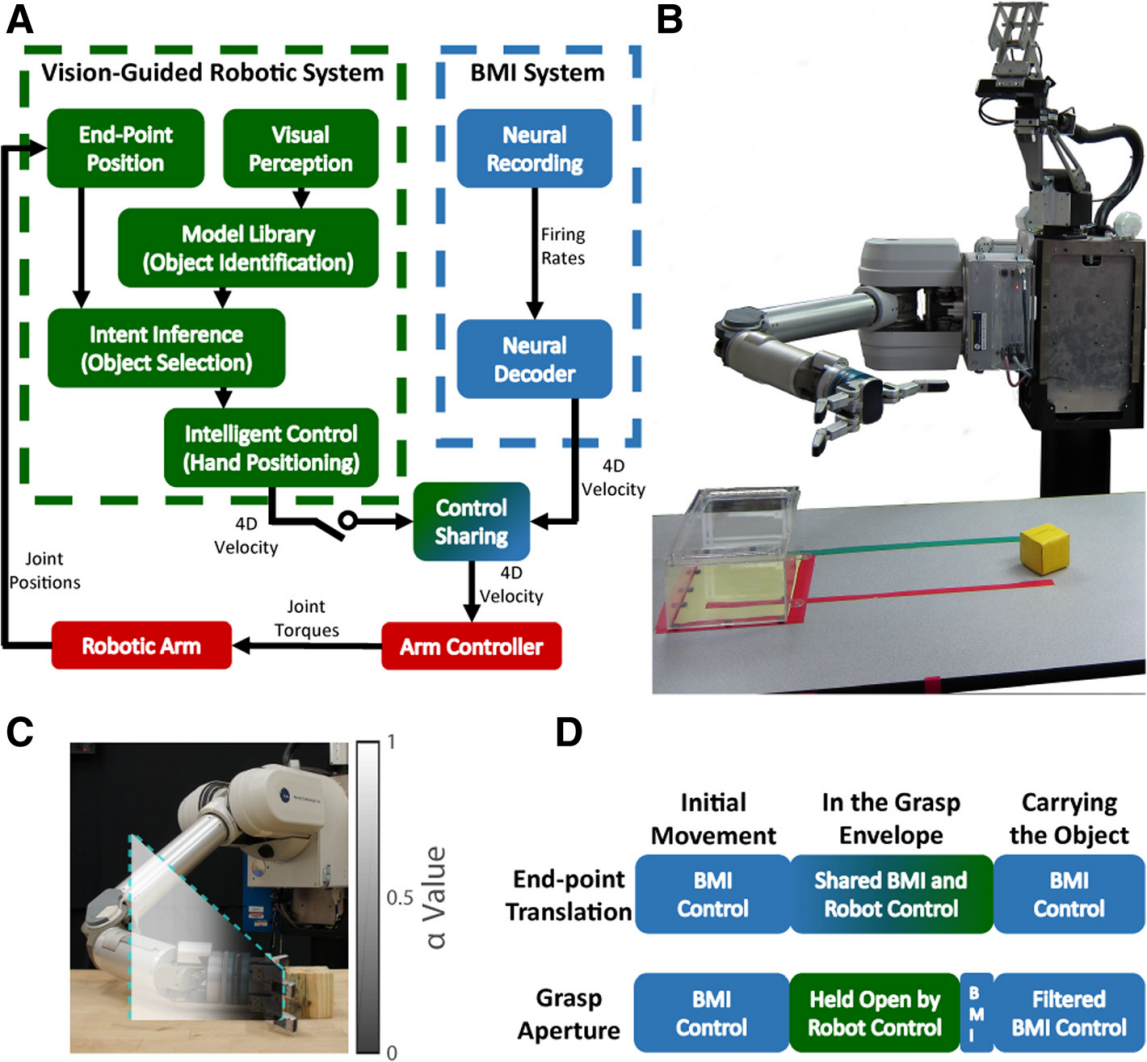
Shared control system diagram and robot testing set up. **a** System diagram for the vision-guided shared control. The *blue boxes* show the BMI system decoding endpoint translational and grasp velocity. The *green boxes* show the components of the vision-guided robotic system for grasping. If shared control was not in use, only the output of the BMI system was used to send commands to the arm, but with shared control, the control signal of the vision-guided system was blended with that of the BMI system to create the final robot command. **b** The 7.5 cm cube (*yellow*) and the target box (*clear box*) were positioned on the table, as shown, to start the ARAT trials. The subject sat approximately 1 m to the left of the robot. **c** An example of the central cross-section of the grasp envelope for a stable grasp position on a 7.5 cm cube is outlined by the *blue dotted line*. The shading shows the gradient of shared control (α value), with white areas being completely controlled by the BMI user and darker areas having more robot control. **d** A trial progression schematic showing when translation and grasp control are under BMI control (*blue*) or robot control (*green*). Wrist orientation was always maintained in a neutral posture under computer control

The goal of the experiment was to enable BMI control of a robotic arm (WAM Arm, Barrett Technology, Inc., Newton, MA) during reaching and grasping tasks. The WAM Arm is a 7 degree of freedom robot with a 4 degree of freedom 3-fingered Barrett Hand (Fig. 2b). Each day a neural decoder was created to transform neural firing rates into continuous 3 dimensional endpoint translation and 1 dimensional grasp velocity commands for the WAM Arm. The wrist was maintained in a neutral position by computer control during calibration and throughout testing. Custom code was used to compute joint torque commands to execute the decoded endpoint velocity commands.

A two-step calibration method was used to create an optimal linear estimation (OLE) decoder as previously described [2, 3]. The subjects first observed and attempted to control the WAM Arm as the computer commanded it to move. Each trial started with translation to one of 10 possible targets (points in space) at which point the hand grasped or released. Subject 1 completed 40 trials, while Subject 2 completed 60 trials, taking approximately 10 or 15 min respectively. Once this step was completed, an OLE decoder was derived based on an encoding model relating recorded firing rates to the computer - generated arm and hand kinematics. The encoding model relating unit firing rate to arm kinematics is:

$$ f={b}_0+{b}_x{v}_x+{b}_y{v}_y+{b}_z{v}_z+{b}_g{v}_g $$

where *f* is a unit’s square root transformed firing rate, *v* is the kinematic velocity and *b* is the calculated coefficient relating the velocity and firing rate for each controlled dimension. The dimensions are *x*, *y*, and *z* translation and *g* grasp aperture. Optimal linear estimation with ridge regression was used to transform calculated b coefficients in to decoding weights that were applied to recorded firing rates to generate kinematics for the arm [2].

The decoder trained in this first step was then used during a second step to give the subjects control of the arm in order to execute the same task as in the first stage of calibration. During this second step the computer restricted decoded brain control commands to those that moved the hand directly towards or away from the specified target during the translation phase of the trial as demonstrated by Velliste et al. [24]. The arm position was held still while the user issued grasp velocity commands during the grasp phase of each trial. A new OLE decoder was trained using the recorded firing rates and kinematics generated during 40 (Subject 1) or 60 (Subject 2) reach and grasp movements under brain control. The gains for translation and grasp velocity commands were tuned with feedback from the subjects to achieve what they felt was the best balance of speed and accuracy when using the BMI alone (i.e. without shared control). This new decoder was then used for BMI control during the rest of the testing session (Fig. 2a, blue blocks).

### Vision-guided robotic shared control

Shared control was provided by utilizing a computer vision system that monitored the work environment with a RGB-D camera mounted above the arm base (Fig. 2b). The vision system identified objects by matching depth-image templates from a model library. This library also contains a set of pre-defined hand positions relative to the object that would result in a stable grasp and each is associated with a grasp envelope. The stable grasp positions for each test object were manually determined prior to testing. The grasp envelope was a 25 cm long truncated cone with the small end being the size of the object. The grasp envelope cone opened at an 80° angle from the stable grasp position and was oriented along an ideal approach path for a given grasp position (Fig. 2c).

During the experiment, the subjects controlled the robotic arm and hand to perform reaching and grasping tasks. Once the subject directed the hand into the envelope of a stable grasp of an object, the shared control system inferred that the user intended to grasp that object (Fig. 2a, green blocks). The certainty of this intention was updated for every velocity command from the BMI system (33 Hz for Subject 1, 50 Hz for subject 2). Once the hand was inside the grasp envelope, the system assisted the user in positioning the robot hand to grasp the object by blending the translation commands of the vision-guided robotic system and the BMI system. While the hand was in the grasp envelope, but not yet at the stable grasp position, the hand was maintained in an open position and user generated grasp velocities that would otherwise open and close the hand were ignored. As the hand moved closer to the stable grasp position and the certainty of intention to grasp increased the robotic system’s commands gained more weight, however if the user directed the hand outside the envelope at any point they regained complete control of the arm. Essentially the shared control system regulated the amount of assistance based on the certainty of the BMI user’s intention (Fig. 2a, Control Sharing). The final translation velocity commands sent to the robot were calculated using a linear blending of user and robot-generated commands:

$$ C=\left(1-\alpha \right)R+\alpha B, $$

where *C* is the velocity command sent to the robot, *R* is the robotic system’s velocity command, and *B* is the BMI system’s velocity command derived from the BMI user. The arbitration factor *α* defines the amount of control given to the user and is computed as:

$$ \alpha ={\left(1+{e}^{- aD+o}\right)}^{-1}, $$

where *D* is the scaled distance between the hand and the optimal position for grasping projected along the central axis of the grasp envelope. *D* is 0 when the hand is at the stable grasp position and 1 when the hand is at the furthest point in the grasp envelope. The constants *a* and *o* are parameters that are set to ensure *α* is in the range of [*α*_*min*_, 1]. For this study they were manually set to *a* = 11.6 and *o* = 6.9, making *α*_*min*_ = 0.001. Outside the grasping envelope the user has full control of the robot arm (*α* = 1.0). Once the hand reached the stable grasp position, the robotic system had nearly complete control of hand position (*α* = 0.001). At the grasping pose the user was required to issue hand closing-velocity commands to grasp the object. The object was grasped with a pre-programmed constant finger torque until a release command was issued.

Once a successful grasp was assured by the system, the user regained unassisted control of endpoint translation while the assist system applied a low-pass filter to the grasp velocity commands, so that transient release commands did not cause the object to drop. The low-pass filter was applied until the hand reached the pre-programmed release area or the subject issued a prolonged hand-opening velocity command to release the object. Figure 2d provides a timeline view of when robotic assistance or BMI control were responsible for translation and grasp velocities. A more detailed description of the vision-guided robotic shared control system is given in Muelling et al. [25]. This previous conference paper focused on the technical details of the system and included high level performance metrics, but no detailed kinematic analysis, for 2 of the 5 ARAT sessions in this paper. Muelling et al. [25] also described the results of more unstructured tasks that demonstrated the abilities of the system but did not allow for comparison to unassisted control.

### Action Research Arm Test

Functional control of the robotic arm was tested using a subset of the Action Research Arm Test (ARAT) that was developed for measuring arm and hand function during recovery from stroke [22]. The ARAT involves moving cubes of various sizes from one location to another. For each ARAT trial, the cube (2.5, 5, 7.5, or 10 cm) was placed on the left side of the table, approximately 40 cm to the left of the hand’s starting position which was 30 cm above the table, similar to [2, 3] (Fig. 2b). The subject was instructed to pick up the cube and place it on top of a box positioned on the right side of the workspace. The subject could regrasp an object if it was dropped or moved. Completion times were recorded for successful trials. A trial was marked as out of bounds if the object was pushed or dropped outside the workspace of the arm. All other trials ended after 2 min and were marked as timed out. Subjects were informed that all 3 trials would be counted towards their performance score, in contrast to previous studies which instructed participants that only the best trial would count, allowing for different approach strategies to be tested [2, 3]. Trials were presented in blocks of 3 with the same cube size and assistance conditions (with or without shared control). The subject was not told which condition was being tested and the order of the test conditions was randomized. After each block the subjects were asked to rate the difficulty of the task on a scale of 1 to 10 (1 = easiest, 10 = hardest task imaginable). Subject 1 completed 3 sessions of ARAT testing, and Subject 2 completed 2 sessions.

### Trajectory comparisons

To identify the effect of shared control on the kinematics of the ARAT we analyzed the path lengths of successful trials. Here we consider path lengths for the whole trial, for before the first grasp attempt, and for after the object was successfully grasped for the last time. The first grasp attempt was identified as the initial decrease in aperture to less than 10 % of the full range. The path length before this point was labeled as pre-grasp path length. In order to calculate post-grasp path length, we identified the last grasp (10 % of the minimum aperture) prior to a successful release of the object. This allowed us to compute a post-grasp path length even when the object was dropped during the transport phase. When only one grasp was required to complete the trial the path length for the whole trial was equal to the pre-grasp path length plus the post-grasp path length, but in all other cases the sum of the two would be smaller than the whole trial path length.

### Multiple object task

**Fig. 3:**
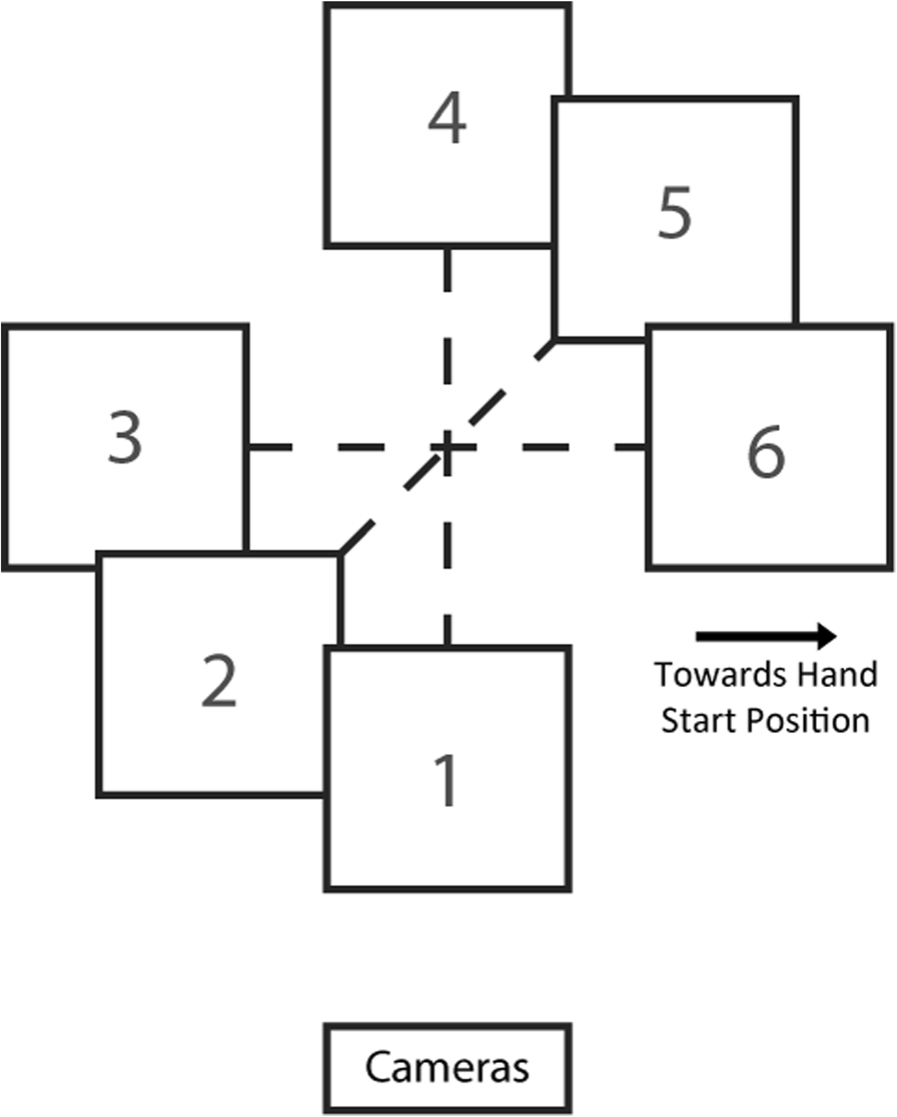
Target positions for the multiple object task. The 7.5 cm target cubes filled the squares in the diagram and were separated by 10 cm. For a single trial, the cubes were placed at 2 positions connected by *dashed lines*, and the subject was instructed to pick up 1 of the 2 cubes. The position numbers correspond to the target numbers in Table 2. The cube in Fig. 2b is at the same point on the table as the intersection of the dashed lines here. The “Cameras” *box* and hand position *arrow* indicate the location of those components of the robot at the start of the trial

After completing both sessions of the ARAT task, Subject 2 was asked to perform a task that required him to correctly select one of two objects placed on a table. Subject 1 was no longer participating in the study by the time this task was developed. At the beginning of each trial, two 7.5 cm cubes were placed 10 cm apart (from inside edge to inside edge) in one of 3 possible orientations (Fig. 3). The subject was told which of the two objects to grasp immediately prior to the trial starting, but the robotic system was not informed of the target. The goal of the task was to select the correct cube and lift it at least 7.5 cm off the table (i.e. above the other cube). This task was in some ways easier than the ARAT since the object only had to be grasped and lifted, but not transported and released at a new target. The subject had 60 s to complete the trial and completion times were recorded for successful trials. In addition to timing out, trials were considered failures if the cube was pushed beyond the reach of the arm, or if the wrong cube was lifted. The test session consisted of 24 trials with assistance and 24 trials without assistance, in randomized blocks of 4–5 consecutive trials under one condition. At the end of each block, the subject reported a 1–10 difficulty score as in the ARAT.

## Results

### Action Research Arm Test performance comparison

**Fig. 4:**
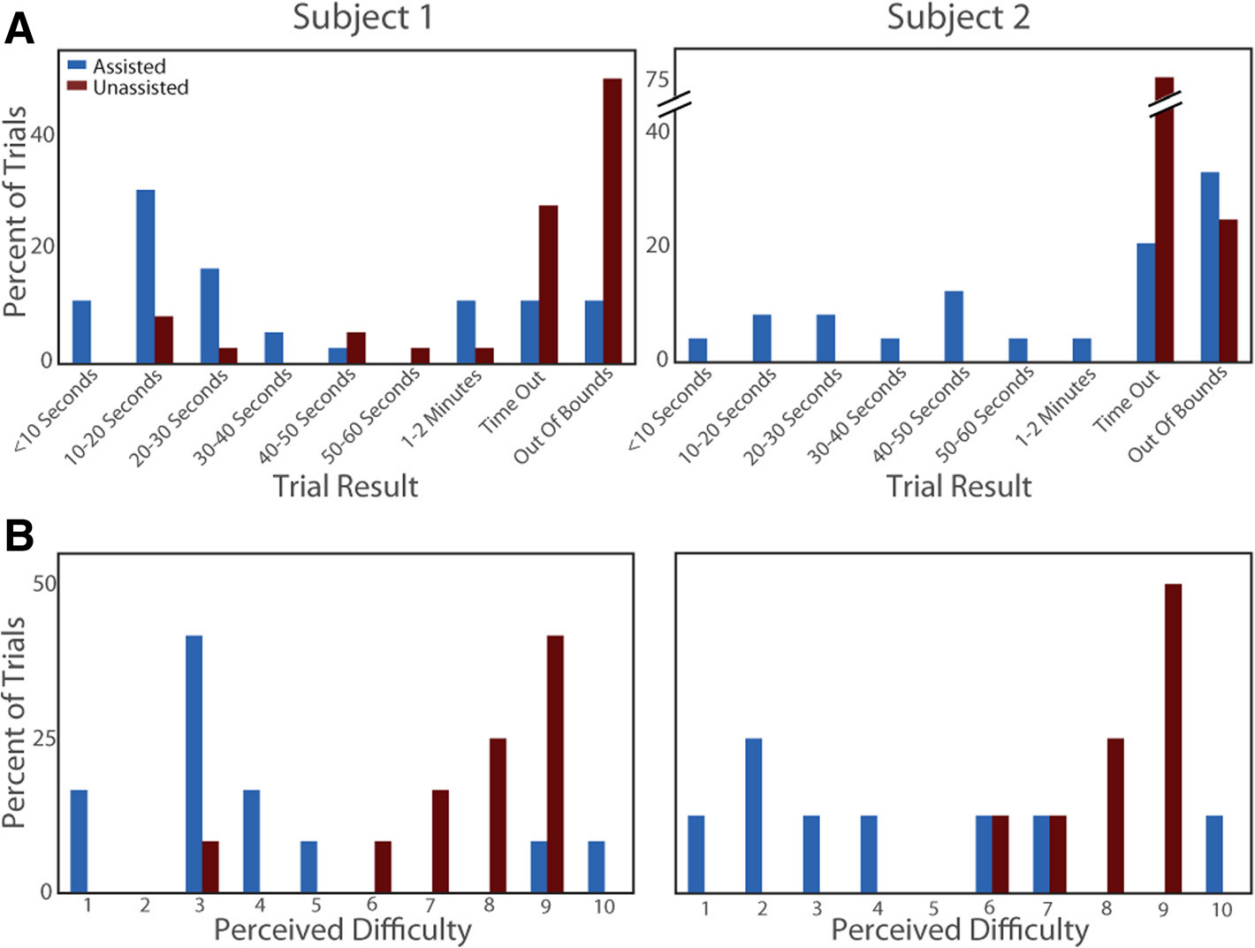
ARAT performance and difficulty. **a** The frequency of each trial result for Subject 1 (*left*) and Subject 2 (*right*). Completion times are shown for successful trials and the failure mode (time out or out of bounds) is noted for failed trials. Assisted (*blue bars*) and unassisted (*red bars*) trials are shown separately. **b** The frequency of each reported difficulty score for assisted and unassisted trial sets (1 = extremely easy, 10 = extremely difficult). Both subjects were more successful and reported that the task was easier during the trials with shared control

Both subjects performed significantly better on the ARAT tasks with shared control (both subjects: *p* < 0.001, Fisher’s Test). Subject 1 successfully completed the tasks in 78 % of the trials with shared control while only succeeding in 22 % of trials without. Subject 2 successfully completed the tasks in 46 % of trials with shared control, but failed all unassisted trials. Figure 4a shows the distribution of completion times for successful trials, as well as the percentage of trials that failed by timing out, or moving the object out of bounds. The median completion time for Subject 1 for trials with shared control was 17.5 s, while unassisted trials had a median of 31.5 s. With only 8 successful unassisted trials, this difference did not reach significance (*p* = 0.31, Wilcoxon rank-sum test). The median completion time for successful shared control trials for Subject 2 was 35 s.

The purpose of using shared control is to make tasks easier, make it possible to perform a wider range of tasks, and to alleviate user frustration. Participants reported a 1–10 difficulty score after each set of three trials with a single cube size and test condition (with or without shared control) combination. Both subjects had an easier experience during shared control trials. Subjects 1 and 2 reported an average difficulty score of 4.1 and 4.4 for assisted trials and 7.7 and 8.1 respectively for unassisted trials (both subjects: *p* < 0.02, Wilcoxon signed-rank test; Fig. 4; Table 1). Success rates, completion times, and difficulty ratings for each cube size are listed in Table 1. Additional file 1: Movie S1 shows the fastest trial for each cube size with and without shared control.

**Table 1:** ARAT performance metrics

		Success rate		Mean completion time (sec)		Mean difficulty	
Sessions	Cube	w/Assist	w/o Assist	w/Assist	w/o Assist	w/Assist	w/o Assist
Subject 1	10 cm	67 %	0 %	25	–	3.7	8.3
	7.5 cm	100 %	44 %	22	23	3.0	6.3
	5 cm	67 %	11 %	16	14	4.7	7.7
	2.5 cm	78 %	33 %	48	64	5.0	8.3
	Total	78 %	22 %	28	37	4.1	7.7
Subject 2	10 cm	0 %	0 %	–	–	7.0	7.0
	7.5 cm	50 %	0 %	29	–	4.5	7.5
	5 cm	50 %	0 %	20	–	4.0	9.0
	2.5 cm	83 %	0 %	42	–	2.0	9.0
	Total	46 %	0 %	33	–	4.4	8.1

Subject 1 completed 9 trials of each cube size, both with and without shared control assistance. Subject 2 completed 6 trials of each. The time to complete the task is averaged across all successful trials. If there were no successful trials the cell was left blank. Mean difficulty is on a 1 (extremely easy)-10 (extremely difficult) scale

### Trajectory comparisons

**Fig. 5:**
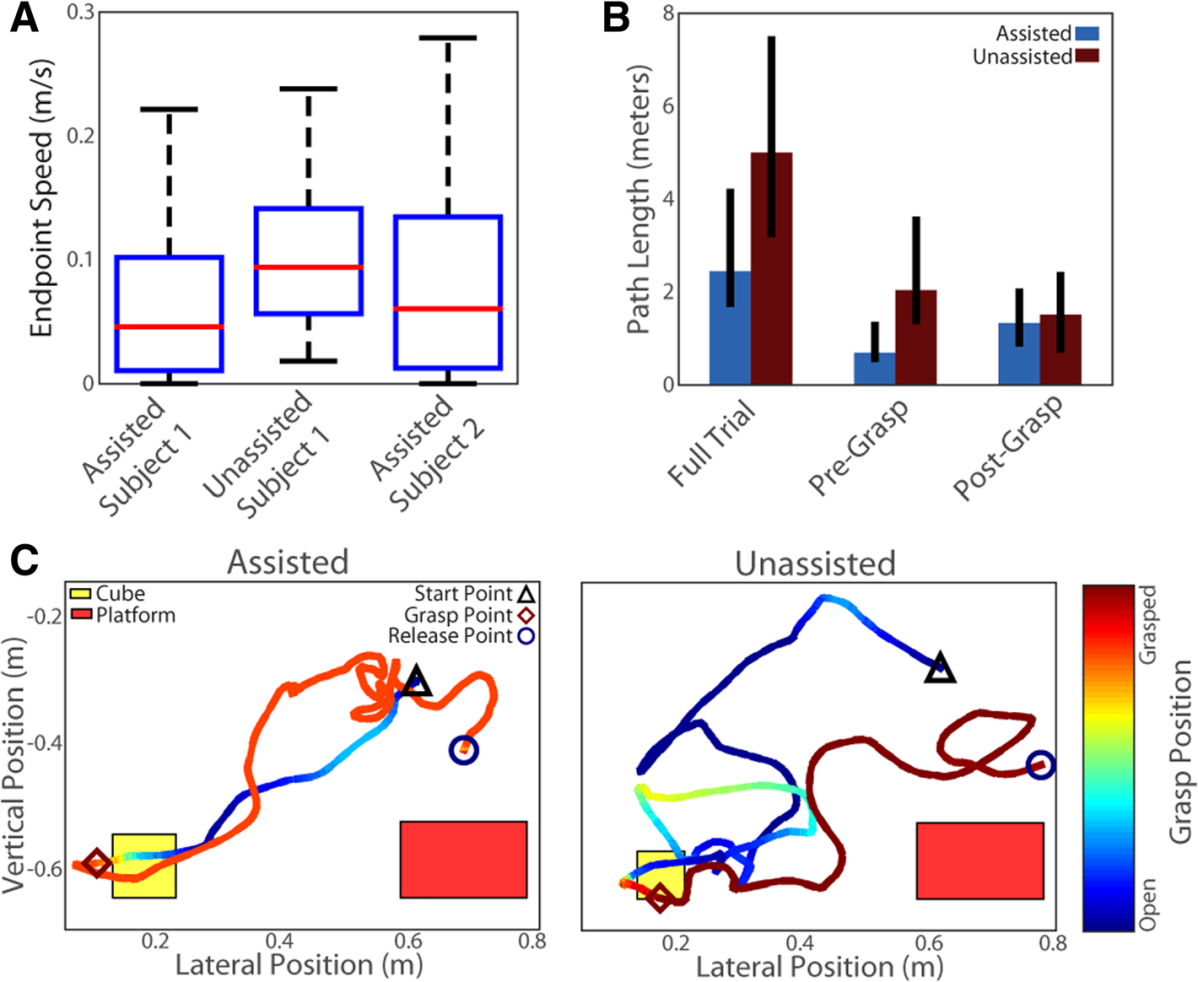
Analysis of trajectory properties with and without shared control. **a** A box plot distribution of hand translation speeds across all time bins while the hand was less than 10 cm above the table during successful trials. The *red line* is the median speed, the *blue box* show the interquartile region, and the *whiskers* span the 5th-95th percentile. The speed distribution for assisted trials for both subjects skews low indicating that the hand was steadier when approaching the object. **b** Subject 1’s path lengths during successful trials, first for the full trials, then separated by the path length before the first grasp attempt and the path length after the object was grasped. Error bars span the interquartile region. The assisted trials benefit the most during the pre-grasp portion of the trial. **c** Subject 1’s hand trajectories with median path lengths for their assistance condition. The color shows the grasp aperture. The release point is marked where the hand opened to allow the object to drop onto the platform. We did not specify to the subject how the object had to be placed, or released, onto the platform. Additional file 2: Movie S2 shows both trials

To identify how the use of shared control affected the execution of the ARAT task, we computed the speed profile and total path length for each trial. Since Subject 1 was the only subject to complete ARAT trials without assistance, her data forms the primary basis of comparison shown in Fig. 5. Endpoint speed for Subject 2’s assisted trials is shown for reference. Specifically, we show the distribution of endpoint speeds when the hand was within 10 cm of the table during successful trials for both subjects (Fig. 5a). We used this distance criterion to identify hand approach, even if the subject had difficulty grasping, or bumped the object before successfully grasping it. The distributions of endpoint speed show that lower hand speeds are maintained during trials with shared control as compared to unassisted trials.

As shown in Fig. 4 and Table 1, Subject 1 tended to have faster trial completion times when using shared control (but did not reach significance). The faster completion times during shared control trials, which also had slower movement speeds, indicate that movements were more direct. The direct movements are apparent from Subject 1’s significantly shorter overall path lengths during trials with shared control (median: 2.44 vs. 5.00 m, *p* = 0.026 Wilcoxon rank-sum test, Fig. 5). While variability in task execution was present between trials, Fig. 5c shows the endpoint and grasp trajectories for the trials nearest the median in overall path length to provide a representative comparison of performance between the two conditions.

To identify which phase of the task was most improved by shared control, we examined the path length before and after the first grasp attempt of each trial, defined as when the grasp aperture first reached 10 % of its minimum in each trial. Note that the subject missed the block on the first try and had to grasp again on 7 % of assisted and 25 % of unassisted successful trials. On these trials with missed grasps 2–4 total grasp attempts were made. She also dropped the block after grasping it but before reaching the release point on 21 % of assisted and 13 % of unassisted successful trials and needed to grasp it again to finish the trial. On trials in which the cube was dropped there were typically only 2 total grasp attempts. In both cases, movements after the first grasp attempt were not included in the calculation of pre-grasp path length. Likewise, movements before the final grasp attempt were not included in the calculation of post-grasp path length. Shared control trials had much shorter path lengths prior to grasp (median: 0.70 vs. 2.04 m, *p* < 0.005 Wilcoxon rank-sum test), while the difference was less pronounced during the carry phase, which occurred after the object was grasped for the last time (median: 1.34 vs. 1.51 m, *p* = 0.86 Wilcoxon rank-sum test). The shorter path lengths and slower hand movements prior to grasping indicate that the shared control improved task performance primarily by stabilizing the movement near the object to eliminate unintended or inaccurate movements that could interfere with the user’s ability to quickly complete the task (Additional file 1: Movie S1 and Additional file 2: Movie S2).

During successful shared control trials, some level of computer assistance was active for 49 % of the trial on average (Subject 1 – 53 %, Subject 2 – 38 %). This assistance was primarily rendered while the user was attempting to grasp the object, which takes a great deal of precision, making up 34 % of the total trial time. After grasping, the subjects regained complete control of translation and only grasp aperture was assisted to aid with carrying of the object. The object was grasped for transport for 15 % of the trial time on average including the multiple transport attempts if the object was dropped prematurely. This amount of time is small relative to the time to grasp the object because releasing the object required much less precision than grasping it. The subjects were in complete control of the arm for 51 % of the trial on average.

### Selection from multiple objects

Subject 2 successfully lifted the correct object in 92 % of trials with shared control compared to only 46 % of unassisted trials during the multiple object task (Table 2; significantly different with *p* < 0.001, Fisher’s test). Only 2 of the 24 trials with shared control were failures, one for lifting the wrong object, and one for moving the target out of the workspace without lifting it off the table. In contrast, 13 of the 24 trials without shared control were failures, 8 because the targeted object was moved outside of the arm’s workspace, and 5 because the targeted object was not lifted within the 1 min time limit.

**Table 2:** Multiple Object Task Performance Metrics

	Success rate		Median completion time (sec)	
Target	w/Assist	w/o Assist	w/Assist	w/o Assist
1	100 %	50 %	8.7	19.3
2	100 %	50 %	8.4	17.8
3	75 %	67 %	7.7	30.5
4	100 %	80 %	8.1	20.7
5	100 %	25 %	8.3	28.7
6	75 %	0 %	9.6	–
Total	92 %	46 %	8.3	26.3

Both success rate and median completion time for successful trials are improved with shared control for all target positions (as numbered in Fig. 4) from the multiple object task performed by Subject 2

In addition to increasing the frequency of successful trials, shared control decreased the median completion time of successful trials from 26.3 s to 8.3 s (Table 2; *p* = 0.03, Wilcoxon rank-sum test). Consistent with the clear difference in performance, the subject gave the shared control trials an average difficulty rating of 1.4 and the unassisted trials a difficulty rating of 5.6. The fastest trials at 4 of the 6 target positions with and without shared control are shown in Additional file 3: Movie S3.

## Discussion

In this study, we showed that the combination of a BMI system blended with vision-guided autonomous robotic control, improved the operation of a robotic arm during moderately complex reach-and-grasp tasks. Once computer vision had identified objects in the workspace, the shared control algorithm used the observed BMI-generated arm trajectory, to infer intention and compute a new command signal with the appropriate contributions from BMI and autonomous control. Previous studies that combined BMI control with a computer vision-based autonomous robotic system relied on visual attention toward a specific object on a computer screen. One study identified targeted objects on a computer display using gaze tracking [14] and another, relied on the EEG P300-evoked response to flashes on the screen near objects [13]. In both cases, once the object was selected, the task was completed automatically. The user was unable to intervene. Rather than relying on triggered pre-programmed arm movements, our system continuously tracked objects while the user maintained high-level control of the arm from which the shared control system could infer intent. Shared control allowed the system to continuously correct for user errors, such as dropping an object in a new location, and allowed the user to correct system errors, such as incorrectly identifying the desired target (Additional file 4: Movie S4). Our objective was to maintain as much volitional control as possible, while providing assistance for the most difficult parts of the task. Once the shared control system detected that the user had selected an object, the hand was stabilized for grasping. Closure of the hand around the object was triggered by the subject. This method left the users in control of the task progression at all times and allowed them to override the assistance by moving the arm away from the object if the robotic system misidentified the user’s intent (Additional file 4: Movie S4).

There have been two published shared control systems involving intracortical BMI in a human subject [15, 16]. Katyal et al. presented a system that gave a BMI user translational control until the hand approached a visually identified object at which point entirely autonomous translation and grasping was triggered [16]. This system only dealt with one object, and automatically grasped the identified object based on hand proximity with no attempt to identify intent and did not allow the subject to abort the grasp. In a different study by Vogel et al., the subject controlled 2 degrees of arm translation and a separately decoded discrete state switch command [15]. When the state switch command was detected, the system executed a pre-programmed drinking task (i.e., grasp and lift bottle, tilt bottle, or lower and release bottle) and waited for the next state switch command. When attempting to grasp or move the bottle, the user had unassisted control of the 2 degrees of translation. While these systems worked well for specific pre-programmed tasks, they could not generalize to novel settings or adapt to real-world use.

For the ARAT trials, the users maintained complete control of the arm for the majority of the trial. While computer assistance was active for less than half of the ARAT trial on average, it shortened the hand’s path length, especially prior to grasping. This demonstrates the significant improvement in the reach-to-grasp movement with shared control. This improvement results in a decreased endpoint velocity when the hand is near the table, indicating that the hand is stable while grasping. Grasping the object while the hand moves quickly might be the ideal result to minimize trial time, however we observed that if the hand moved quickly while near the object it often knocked the object out of position rather than grasping it, making lower velocities near the object more beneficial. We also included a task with multiple objects in order to demonstrate the system’s flexibility in a more realistic environment where the user had to choose the correct target. Subject 2 was much more successful on the multiple object tasks with shared control than without. Additional file 2: Movie S2 shows that he had less unintended contact with the untargeted object during shared control trials than those without. Additional file 4: Movie S4 also shows that he was able to move away from the incorrect object even after the system had identified it as the target. This real-time shared control system accomplished the goals of maintaining user autonomy when possible, limiting user frustration due to undesired movements, and decreasing the overall difficulty of using the system.

Shared control can extend the limits of current BMI technology. One problem with the chronic microelectrode recording technique is the degradation of recorded signal quality over time [5]. This was a factor for Subject 1, who had been able to control the robotic arm with up to 10 degrees of freedom (DoF) [3]. In general, the number of DoF a user can control is limited by the relevant information that can be extracted from a limited sample size of recorded units. Because of the worsening signal quality, 31 months into the study Subject 1 could only control 4 DoF (3D translation and grasp) with variable levels of performance. Shared control compensated for this decline in performance and allowed her to more reliably and easily perform object-transfer tasks with 4 DoF. Similarly, Subject 2 was limited to unreliable 4 DoF control at the time of this study. Despite the limited DoF, both subjects were able to achieve reliable functional control with the shared control system. If control is limited even further than it was for the subjects in our study due to signal quality degradation or limited recordable information, assistance may need to be increased. It may be possible to increase the size of the grasp envelope or to automate grasping completely so that the system is less reliant on the user’s unreliable input. However, this would come at the cost of generalizability to novel tasks. The system would need to be customized to provide optimal performance while maintaining a desirable level of independence for the user.

Another general problem is that BMI users currently rely exclusively on visual feedback, which may contribute to ineffective and unstable grasping [7]. The increased stability from shared control resulted in low endpoint speeds when the hand was in the grasp envelope (Fig. 5). While slowing down or smoothing the translation commands might create similar stability, this would likely come with a tradeoff of slowing other phases of movements or limiting the ability to make corrective movements. To compensate for the lack of somatosensation, the shared control system biased the hand aperture toward a closed configuration once an object was grasped, decreasing the likelihood of the object being dropped prematurely. Shared control is one way to maximize the function of a low dimensional control signal and/or limited feedback.

While this prototype shared control system already demonstrated how the addition of autonomous control can enhance BMI performance, a number of advances can be made in the near future. The object library in these sessions was primarily composed of simple geometric objects, but could be expanded to include a large variety of objects, and together with machine-learning algorithms, used to identify graspable portions of larger objects. Muelling et al. [25] also showed the potential to add pre-programmed actions for a subset of identified objects, such as turning a door knob or pouring from a can, that could provide assistance for higher level tasks. This is similar to the work completed by Vogel et al. [15] with the addition of continuous object identification and shared control. Ideally, this pre-defined information could be replaced by brain-derived signals encoding object identity and intended use. Further, this system could be modified to allow the user to turn the assistance on and off using brain-derived, or external (e.g. switches), control signals. This would allow the user to more easily interact with objects with different intentions, such as pushing an object versus grasping an object. Additionally, while the release zone for the ARAT task in the current study was specified explicitly in the system, release of an object could be assisted based on proximity to a surface like a shelf or table, allowing generalization to a wider variety of tasks. In future applications, the camera could be mounted to the user’s wheelchair along with the robotic arm to allow for maximum utility and portability.

The work here is a proof-of-concept as an initial step to create a more flexible system to make neuroprosthetic arms more functional for future users. In this study the subjects reported that using the arm was significantly easier with shared control than with BMI alone, and while they recognized when the assistance was active, they never commented that it interfered with their intended actions. Balancing the control between the user and the automated system will be important to provide high performance while ensuring that the user feels that they can use the device reliably in many different situations. As both technologies continue to improve, robotic prosthetic control should become both easier and more useful for the people who need it.

## Conclusions

The combination of BMI and computer vision-based grasping creates a system that can allow people without use of their arms to control a robotic prosthetic to perform functional tasks in cases where neither technology would be sufficient on its own. The BMI provides the user with high-level control of the pace and goals of the arm movements. The computer vision system helps with the details of the movement, ensuring a secure grasp in the presented cases, but also by identifying how to act on a specific object based on its shape [25]. Balancing the control between the user and the automated system will be important to provide high performance while ensuring that the user feels the device is reliable and responsive to their commands in a variety of situations. As both technologies continue to improve, robotic prosthetic control should become both easier and more useful for the people who need it.

## Additional files

Additional file 1: Video 1. A comparison of the best performance by Subject 1 for each object with and without shared control. In all cases except the 10 cm cube without shared control, the best trial was the one with the fastest completion time. The 10 cm cube was never successfully moved without shared control so the best trial was the one that moved the object to the platform even though it was not released properly. (MP4 23394 kb) Additional file 2: Video 2. A comparison of Subject 1’s ARAT trials with the median path length with and without shared control. The comparison shows that shared control made typical successful trials quicker and easier in addition to making more trials successful. (MP4 5982 kb) Additional file 3: Video 3. A comparison of the fastest multiple object trials to targets 1, 2, 4, and 6 with and without shared control. Without shared control the subject was more likely to move the cube that he was not trying to lift, and more likely to drop the target cube immediately after lifting it. (MP4 14176 kb) Additional file 4: Video 4. Two video clips showing the flexibility of the shared control system. In the first clip the subject prematurely drops the cube during the ARAT task. The vision-guided grasping system identifies it on the table and assists with grasping it again once the subject moves into the appropriate workspace, resulting in a successful trial. In the second clip, the vision-guided grasping system incorrectly determines that the subject wants to grasp the wooden cube instead of the yellow one. The subject is able to move the hand away from wooden cube, and after doing so the system correctly identifies the subject’s intent to grasp the yellow cube. (MP4 11324 kb)

## Abbreviations

ARAT: Action Research Arm Test
BMI: Brain-machine interface
DoF: Degrees of freedom
EEG: Electroencephalography
OLE: Optimal linear estimation
RMS: Root mean square
